# GenPhyloData: realistic simulation of gene family evolution

**DOI:** 10.1186/1471-2105-14-209

**Published:** 2013-06-27

**Authors:** Joel Sjöstrand, Lars Arvestad, Jens Lagergren, Bengt Sennblad

**Affiliations:** 1 Department of Numerical Analysis and Computer Science, Stockholm University, Stockholm, Sweden; 2 Department of Medicine, Karolinska Institutet, Atherosclerosis Research Unit, Stockholm, Sweden; 3 School of Computer Science and Communications, KTH Royal Institute of Technology, Stockholm, Sweden; 4 Science for Life Laboratory, Stockholm, Sweden; 5 Swedish e-Science Research Centre, Stockholm, Sweden

**Keywords:** Phylogenetics, Synthetic data, Gene family, Gene duplication, Gene loss, LGT, Molecular clock, Biogeography, Host-parasite co-evolution

## Abstract

**Background:**

PrIME-GenPhyloData is a suite of tools for creating realistic simulated phylogenetic trees, in particular for families of homologous genes. It supports generation of trees based on a birth-death process and—perhaps more interestingly—also supports generation of gene family trees guided by a known (synthetic or biological) species tree while accounting for events such as gene duplication, gene loss, and lateral gene transfer (LGT). The suite also supports a wide range of branch rate models enabling relaxation of the molecular clock.

**Result:**

Simulated data created with PrIME-GenPhyloData can be used for benchmarking phylogenetic approaches, or for characterizing models or model parameters with respect to biological data.

**Conclusion:**

The concept of tree-in-tree evolution can also be used to model, for instance, biogeography or host-parasite co-evolution.

## Background

Software that simulates synthetic trees has long been an important tool in phylogenetics for investigating tree distributions of studied models, or for evaluating performance of various inference methods. The traditional approach has been to generate trees using a birth-death model (e.g., [1,2]). In population genetics, generation from the coalescent model of allele evolution is central, and several allele tree generators exist; some allowing constraints by a population genealogy [3,4], while others are unconstrained (e.g., [5]).

We present GenPhyloData: a suite of tools in the PrIME software project based on various probabilistic models of gene evolution used in other applications in PrIME [6-12]. Phenomena modeled by PrIME include gene duplication, gene loss, lateral gene transfer (LGT), clock models, and sequence evolution. The main feature of GenPhyloData is the ability to simulate tree evolution guided by another tree. The classical example is the evolution of genes through speciations, duplications, losses and LGT events over a species tree, producing a gene tree [10,13], but there are several other applications: (i) Species evolution inside an area tree representing relationships of geographical areas through allopatric and sympatric speciations and migration events. (ii) Parasite evolution within a host tree through co-evolution, independent parasite speciations and host-switching. (iii) Evolution of protein domains within a gene tree through gene duplications, domain duplications and recombination.

Other variants can be envisioned. We will, therefore, use the general nomenclature of a *guest tree* (e.g., a gene tree) evolving inside a *host tree* (e.g., a species tree).

## Implementation

**Figure 1:**
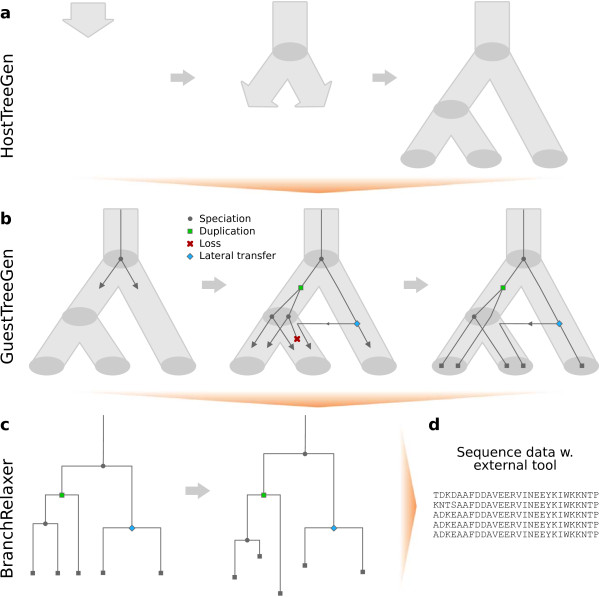
**Illustrates a typical workflow with GenPhyloData for generating a synthetic gene family.** (**a**) A species tree is generated through a birth-death process over a given time interval. (**b**) A gene family is produced by letting an initial lineage evolve down to the leaves of the species tree by means of duplication, loss, lateral transfer and speciation events. The final gene tree is pruned of lost lineages. (**c**) The branch lengths of the clock-like gene tree are relaxed. (**d**) A multiple sequence alignment is created by applying a model of molecular sequence evolution over the relaxed gene tree.

GenPhyloData is implemented in Java. In its current invocation, the suite comprises three tools that may be used in conjunction or separately, as desired (Figure 1):

A. *HostTreeGen*: Generates a bifurcating tree under a birth-death process.

B. *GuestTreeGen*: Given a dated host tree—biological or from (A)—generates a bifurcating guest tree evolving over the host tree by means of duplication, loss and lateral transfer events.

C. *BranchRelaxer*: Relaxes the clock-like branch lengths of a tree—for instance one created with (B) or (C)—by rescaling the branch lengths in accordance with a relaxed clock model.

As an additional fourth step, one can input the simulated Newick trees to a sequence-generating application (e.g., [14,15]) to produce synthetic sequence data in agreement with a suitable model of molecular sequence evolution; see Figure 1d.

## Results

Below, we first introduce the relevant models, and then describe the three tools that implement them.

### The birth-death process

The birth-death process is a well-characterized mathematical model [16], used in biology in general, and in evolution in particular [17,18]. In the context of host tree evolution, the birth-death process over a pre-defined time interval can be described by the following properties; see Figure 1a: (i) A single lineage starts evolving at the start of the interval. (ii) At any time, a lineage evolves independently of all other lineages. (iii) For a lineage, births occur at rate *λ* and deaths occur at rate *μ*. A birth creates two new independent child lineages replacing the parental lineage. A death stops the evolution of the lineage. (iv) When a lineage reaches the end of the interval, its evolution stops. The result can be depicted as a bifurcating tree, where edges represent lineages and vertices represent births and lineage ends. Usually, lineages not reaching the end of the interval are pruned away so that leaves of the resulting host tree comprise only extant lineages.

A special instance of the birth-death process is the pure birth process (or Yule process), with *μ*=0. The birth-death process has been used to model speciations and extinctions in species evolution; see, e.g., [18]. An extension where each leaf of the host tree, when reaching the end of the time interval, has a fixed probability of being removed from the tree has been proposed [18]. This could be used to model, for instance, species sampling or mass extinction events.

### The duplication-loss model

The birth-death process has also been used to model gene evolution, an idea originating from Nei et al. [19]. The duplication-loss (DL) model [6,8] is a canonical generalization of the birth-death process onto a bifurcating host tree, *S*, instead of a single time interval. The tree *S* is dated so that vertices have divergence times (and edges thus time intervals). The root of *S* has an additional incoming “stem edge”, representing how long before the root the DL process started. The process can be described by the following properties: (i) The process starts with a single guest lineage at the start of the stem edge. (ii) Over any edge *e* in *S*, guest lineages evolve independently according to a birth-death process with duplication rate *λ* and loss rate *μ*. If a duplication occurs, the guest lineage splits into two child lineages that continue to evolve in *e*. If a loss occurs, the lineage terminates. (iii) When a guest lineage reaches an interior vertex *x* in *S* (i.e., a speciation in a species tree), the lineage splits into two lineages: one evolving in the first child edge of *x*, and the other evolving in the second child edge of *x*. (iv) The process continues recursively to the leaves of *S* where the process stops. The result is a bifurcating guest tree embedded in *S*. Lineages that failed to reach the leaves of the *S* are usually pruned away so as to produce a pruned guest tree.

The DL model was developed to model gene family evolution, but may be used also to model other tree-in-tree evolutionary processes, such as allopatric and sympatric species evolution in area trees [7]. In the current model, *λ* and *μ* are homogenous over the host tree. Support for, for instance, varying *λ* and *μ* over host tree edges, may be included in the future.

### The duplication-loss-transfer model

The duplication-loss-transfer (DLT) model [10] is an extension of the DL model to include lateral gene transfer (LGT; also known as horizontal gene transfer, HGT), an important evolutionary process in, for example, bacteria. The DLT model is analogous to the DL model, save for the addition of LGT events, see Figure 1b. More specifically, over any edge in the host tree, guest lineages are—apart from duplications and losses—also exposed to LGT events at rate *τ,* in the same vein as rates *λ* and *μ*. If a guest lineage has an LGT event in host edge *e*, the lineage will split into two child lineages: one copy will continue evolving in *e*, whereas the other lineage is instantaneously transferred to a uniformly selected contemporaneous host edge *f* ≠ *e.* The transferred lineage continues its evolution in *f*.

Again, while originally developed for gene evolution [10], the DLT model may be used for other tree-in-tree processes, such as modeling biogeography or parasite co-evolution with host-switches. In the current model, *τ* is homogenous over the host tree. It is possible that future versions will refine on this, for instance to allow increased transfer rates between more closely related host edges.

### Relaxed clock models

The notion of a relaxed evolutionary clock for substitutions dates back to Gillespie [20], who, after showing the inadequacy of the molecular clock hypothesis, discussed variants of relaxing it. The essential idea is to multiply the clock-like edge times of a tree with different edge rates provided by a model to achieve relaxed edge lengths (Figure 1c). Two main variants of relaxed clock models exist.

In the first variant, *uncorrelated models*[11,21-23], substitution rates on different edges of the tree are modeled typically as independent and identically distributed (IID) variables. Several underlying distributions have been suggested and evaluated, including the log-normal, gamma and exponential distributions.

In the second variant, *autocorrelated* models [22-26], a gradual rate evolution of the tree is modeled from the root to the leaves, so that rates are autocorrelated over paths in the tree. Different flavors exist, such as using log-normal, Ormstein-Uhlenbeck or CIR distributions for the process.

A different approach to uncorrelated clock relaxation specific to gene trees was taken by Rasmussen and Kellis [27]. This model assumes that the gene tree is reconciled with a species tree. Here the species tree has an individual gamma distribution associated with each species edge, yielding a “species-specific” factor. A gene lineage will derive its rate from the species edges it passes over, multiplied by a “gene family-specific” factor to account for rate heterogeneity across gene families.

We now present the tools that correspond to the models introduced above.

### The HostTreeGen tool

HostTreeGen generates a bifurcating host tree over a specified time interval using a birth-death process; see Figure 1a. The user provides the parameters (*λ*, *μ*) and the time interval for the model. A uniform sampling probability for each leaf of the resulting pruned tree can be applied. It is also possible to incur limits for the minimum/maximum number of leaves in the tree, enforcing an immediate branching at start, sampling of desired pruned tree sizes from a specified list, etc. The standard output consists of both the unpruned and pruned version of the tree (in Newick format), and files with auxiliary information on node counts, time span, etc.

### The GuestTreeGen tool

GuestTreeGen generates a dated guest tree over a dated, clock-like host tree using either the DL model or the DLT model (see Figure 1b). The user provides the host tree and parameters (*λ*, *μ, τ*) for the model. Similar to above, a guest leaf sampling probability can be incurred, along with various options such as minimum/maximum number of guest leaves in total or per host leaf, and sampling the desired size of the pruned guest tree from a list. The output is a reconciled guest tree (in Newick format), both in its unpruned and pruned form, along with a file detailing its reconciliation with the host tree. Auxiliary information is also produced, such as counts of the different event types, and maximum likelihood estimates of rates based on the unpruned tree and the number of events, computed in accordance with [28].

### The BranchRelaxer tool

BranchRelaxer applies a relaxed clock model to a tree, typically a guest tree; see Figure 1c. It generates rates for the edges of the tree, and outputs the tree with relaxed edge lengths. Several clock models are implemented: (i) A molecular clock. (ii) Uncorrelated IID models with the following underlying distributions: expontential, gamma, log-normal, normal, and uniform rates [11,21-23]. (iii) Autocorrelated models include an exponential model [26], two variants of a log-normal model [23,24], and the CIR process [22]. (iv) The host tree-guided model of [27], which as input requires the guest tree to be relaxed, the host tree with assigned edge rate distributions, and a reconciliation of the two trees. Currently, this model does not support LGT events. (v) An empirical model that samples rates uniformly with replacement from a predefined list derived from, for instance, an MCMC analysis.

For all models, it is possible to require edge rates to be within a specified range.

## Discussion

GenPhyloData complements other frameworks for simulation of genome evolution (e.g., [29]) by being specifically tailored for tree-in-tree evolution. The tools generate directly—forward in time—from the model process. This ensures that a correct tree distribution from the model is obtained if generation is repeated a sufficient number of times. A potential future development target is to include support for tree sampling conditioned on, for example, size constraints. This would enable quicker sampling when the desired requirements and model parameters are discordant.

The nature of GenPhyloData makes it particularly well suited for evaluating more refined models of gene family evolution—very much the current progression of phylogenetics. The PrIME package itself contains several applications for parameter inference of some of the models described here [6,10,12], and they have been evaluated using GenPhyloData.

Future extensions of GenPhyloData could include, for instance, modeling of whole-genome duplications (WGDs), duplication or loss rate heterogeneity over the host tree, or models that account for rate changes implied by phenomena such as pseudogenization and neo-/subfunctionalization following gene duplication [30].

## Conclusions

We provide GenPhyloData: to our knowledge the first generator that simulates guest tree evolution over a host tree by means of duplication, loss and lateral transfer events. It enables construction of, and sampling from phylogenetic tree distributions from biologically relevant models. GenPhyloData can be used to characterize parameters for, for instance, gene family expansion in comparative genomics studies, but the software is also suitable for benchmarking phylogenetic reconstruction methods or modeling general tree-in-tree evolutionary processes. GenPhyloData is free software, platform independent, and a tutorial with usage examples is available at its project home page.

## Availability and requirements

**Project name:** PrIME-GenPhyloData

**Project home page:**http://code.google.com/p/jprime/

**Operating system(s):** Platform independent.

**Programming language:** Java.

**Other requirements:** Java 1.6 or higher.

**License:** New BSD License.

**Any restrictions to use by non-academics:** None.

## Competing interests

The authors declare that they have no competing interests.

## Authors’ contributions

JS implemented the software, partly based on an earlier version implemented by BS. All authors contributed to the design of the models, and read and approved the final manuscript.
